# Advances in Artificial Intelligence for Glioblastoma Radiotherapy Planning and Treatment

**DOI:** 10.3390/cancers17233762

**Published:** 2025-11-25

**Authors:** Reid Master, Nesha Rubin, James Sampson, Kamlesh K. Yadav, Shruti Pandita, Aria Sabbagh, Anika Krishnan, Patrick J. Silva, Kenneth S. Ramos, Vincent Gregoire, Nikos Paragios, Sunil Krishnan, Tej K. Pandita

**Affiliations:** 1 School of Engineering Medicine, Texas A&M Institute of Biosciences and Technology, Houston, 77030 TX, USA; reidmaster12@tamu.edu (R.M.); jamessampson@tamu.edu (J.S.); kamlesh.yadav@tamu.edu (K.K.Y.); 2 Center for Genomics and Precision Medicine, Texas A&M Institute of Biosciences and Technology, Houston, 77030 TX, USA; nsr654@tamu.edu (N.R.); ricksilva@tamu.edu (P.J.S.); kramos@tamu.edu (K.S.R.); 3 Department of Biomedical Sciences, College of Osteopathic Medicine, University of Northern Colorado, Greeley, 80639 CO, USA; 4 ICON Clinical Research, 8307 Gault Lane, San Antonio, 78209 TX, USA; spandit1896@gmail.com; 5 Vivian L. Smith Department of Neurosurgery, UTHealth Houston, Houston, 77030 TX, USA; aria.sabbagh@uth.tmc.edu (A.S.); anikakrishnan18@gmail.com (A.K.); sunil.krishnan@uth.tmc.edu (S.K.); 6 Radiation Oncology Department, Centre Léon Bérard, 69373 Lyon, France; vincent.gregoire@lyon.unicancer.fr; 7 CentraleSupélec, University of Paris-Saclay, 91190 Gif-sur-Yvette, France; nikos.paragios@u-psud.fr; 8 TheraPanacea, 7 Bis Boulevard, Bourdon, 75004 Paris, France

**Keywords:** glioblastoma, artificial intelligence, deep learning, radiotherapy planning, auto segmentation, dose prediction and mapping, machine learning, personalized radiotherapy, multimodal imaging, precision oncology

## Abstract

Glioblastoma is an aggressive central nervous system tumor characterized by diffuse infiltration. Despite substantial advances in oncology, survival outcomes have shown little improvement over the past three decades. Radiotherapy remains a cornerstone of treatment; however, it faces several challenges, including considerable inter-observer variability in clinical target volume delineation, dose constraints associated with adjacent organs at risk, and the persistently poor prognosis of affected patients. Recent advances in artificial intelligence, particularly deep learning, have shown promise in automating radiation therapy mapping to improve consistency, accuracy, and efficiency. This narrative review explores current auto segmentation frameworks, dose mapping, and biologically informed radiotherapy planning guided by multimodal imaging and mathematical modeling. Studies have demonstrated reproducible tumor segmentations with DSCs exceeding 0.90, reduced planning within minutes, and emerging predictive capabilities for treatment response. Radiogenomic integration has enabled imaging-based classification of critical biomarkers with high accuracy, reinforcing the potential of deep learning models in personalized radiotherapy. Despite these innovations, deployment into clinical practice remains limited, primarily due to insufficient external validation and single-institution training datasets. This review emphasizes the importance of large, annotated imaging datasets, multi-institutional collaboration, and biologically explainable modeling to successfully translate deep learning into glioblastoma radiation planning and longitudinal monitoring.

## Simple Summary

Artificial intelligence holds the promise of enhanced glioblastoma radiotherapy by improving segmentation accuracy, incorporating biologically informed mathematical modeling, and integrating radiogenomic data for personalized treatment planning. Deep learning-based auto segmentation can achieve high accuracy with reduced interobserver variability, while tumor growth modeling can enable biologically guided, patient-specific dose mapping. Radiogenomic approaches combine imaging and molecular data to noninvasively predict biomarker status and support individualized therapy. However, clinical translation remains limited by the need for large multi-institutional datasets, interpretability, and standardized validation protocols. Emerging advances, such as adaptive radiotherapy, multimodal data incorporation, and foundation models, offer real-time adaptability and further personalization in glioblastoma treatment.

## 1. Essentials

- Deep learning-based auto segmentation models achieve high accuracy and substantially reduced inter-observer variability in glioblastoma radiotherapy planning (pp. 6–8)
- Biologically informed mathematical modeling integrates tumor growth dynamics with imaging, enabling personalized radiotherapy dose mapping strategies (pp. 8–11)
- Radiogenomic models integrating imaging and molecular data predict status of key biomarkers, supporting non-invasive tumor subtyping and personalized therapy (pp. 11–13)
- Multi-institutional datasets, model interpretability, and standardized validation protocols remain critical barriers to clinical adoption of artificial intelligence-guided radiotherapy (pp. 13–15)
- Recent advances, including adaptive radiotherapy, multimodal integration, and foundation models, enable personalization and real-time adaptability in glioblastoma radiotherapy (pp. 15–17)

## 2. Introduction

Glioblastoma is a highly invasive brain tumor with poor prognosis, which has remained rather static during the past three decades with a median survival of 14 months despite aggressive treatment; its mortality is further emphasized by 5-year survival rates near 5% [1]. Standard first-line treatment for high-grade tumors entails maximal safe resection followed by concurrent temozolomide and radiation therapy (RT) for 3–6 weeks with subsequent temozolomide for an additional 6 months. Tumor treating fields (TTF) therapy, an emerging adjunct modality that delivers alternating electric fields to disrupt mitosis, has shown modest survival benefits in select patients [2,3]. Treatment challenges include tumor heterogeneity and diffuse infiltration, difficulty in defining precise tumor margins, and resistance to standard therapies [1].

The current clinical workflow for glioblastoma management typically begins with a neurological exam and imaging such as MRI or PET/CT scans for initial diagnosis [4]. This is followed by surgical biopsy or maximal safe resection of the tumor and subsequent histopathological and molecular characterization of the tumor and surrounding tissue [5]. Aiming to maximize tumor resection volume while preserving healthy brain function, clinicians utilize tumor visualization and cortical mapping methods during surgery, such as ultrasound, fluorescent dyes, and intraoperative neuroanatomical navigation systems [6]. According to patient age, Karnofsky performance status, and tumor classification, clinicians develop a treatment plan utilizing a combination of temozolomide chemotherapy, palliative care such as corticosteroids, and RT, where RT planning plays a major role.

**Figure 1:**
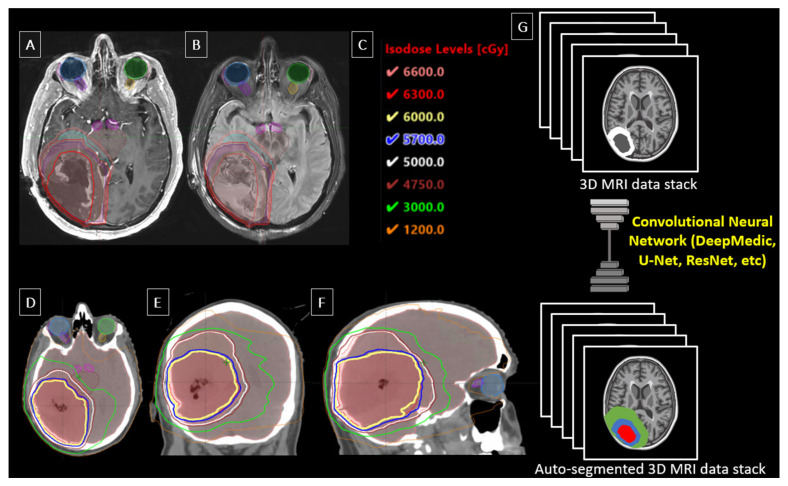
Example segmentation for radiotherapy for a patient with glioblastoma. Contours are drawn in red for GTV outlined on T1c, green for GTV outlined on FLAIR, purple for CTV60 defined as GTVT1c + margin (tucked away from brainstem), outer red for PTV60 defined as CTV60 + 3mm margin, light blue forCTV50 defined as GTVFLAIR + margin tucked away frrom contralateral brain due to falx cerebri serving as an anatomic barrier, and salmon for PTV50 defined as CTV50 + 3 mm margin. (**A**) T1-weighted MRI (axial view); (**B**) FLAIR MRI (axial view); (**C**) Isodose level color key (cGy); (**D**) CT (axial view); (**E**) CT (coronal view); (**F**) CT (sagittal view); (**G**) Pictorial representation of convolutional neural networks in radiotherapy planning for glioblastoma.

The current standard of care for RT in glioblastoma management follows European Organisation for Research and Treatment of Cancer and Radiation Therapy Oncology Group guidelines to generate clinical target volume (CTV), planning target volume (PTV), and gross target volume (GTV) RT dosage maps. European standards require delineation of the T1-weighted contrast-enhancing lesion plus a 2 cm margin, while Radiation Therapy Oncology Group includes FLAIR or T2-weighted abnormalities with a 2 cm margin [7]. An example of a standardized, manually segmented RT dose map for a patient with glioblastoma is shown in Figure 1.

Radiotherapy planning can be conceptually divided into three principal stages: treatment preparation, treatment delivery, and, when required, treatment adaptation.

- Treatment preparation encompasses the delineation of target volumes and organs at risk, the adoption of dose prescriptions, and the determination of treatment plan through simulation.
- Treatment delivery involves the fractionation of the simulated plan into multiple sessions and the systematic administration of radiation according to the established plan.
- Treatment adaptation entails the continuous monitoring of treatment execution and the modification of the plan when anatomical or physiological changes compromise the ability of the initial plan to satisfy predefined dosimetric and clinical constraints.

Because glioblastomas grow aggressively and often infiltrate parenchyma near critical brain structures, accurate tumor delineation is essential for effective RT. Unfortunately, conventional CTV segmentation remains time-consuming and prone to user variability. Artificial intelligence (AI), particularly deep learning (DL) methods such as convolutional neural networks (CNNs), offers an opportunity to improve the accuracy, efficiency, and reproducibility of this process [8]. In simple terms, DL models mimic how the human brain recognizes patterns using synthetic neural nets, which comprise individual synapses, while convolutional neural networks specifically learn visual features from medical images to identify and outline tumor regions more consistently. 

Numerous recent studies have attempted to optimize and automate the radiotherapy planning process [9]. The aim is to enable patient-specific segmentation and dose escalation by leveraging MRI/CT imaging and eventually uncover further dose principles through boosting and local dose control on the basis of radiomics and genetic biomarkers [10].

This review functions as a survey of research publications, exploring recent methodological advances, their progress over the last ten years, current implementations, and future directions. While there are presently several commercial products for tumor auto delineation ready for clinical deployment, a majority of the approaches discussed remain in early adoption stages. For predictive modeling in particular, regulatory certification will prove to be a significant challenge; its early adoption hinges upon extensive clinical evidence with cross-institutional validation. Where possible, the authors highlight whether each study was retrospective or prospective, the size of training/testing data, and testing cohorts. In parallel, we discuss emerging trends for AI in glioblastoma RT planning, including the rise in large-scale foundational models, which will undoubtedly improve inter-observer variability and clinical workflow.

During the last decade, DL models have emerged as a viable solution for the purposes of glioblastoma diagnosis, patient risk stratification, treatment dose escalation, and tumor and organs at risk auto segmentation. Deep learning architecture offers the ability to extract local and global trends among robust datasets without the necessity for quantitative engineering features, which are required for many supervised machine learning (ML) models, such as traditional classifiers or regressors. This review aims to critically evaluate the applications of deep learning in the development of innovative and personalized radiotherapy treatment strategies for glioblastoma, with a particular emphasis on their potential to enhance precision, adaptability, and clinical outcomes.

## 3. Current Challenges in Glioblastoma Management and Treatment

While CNN architectures have dominated the field of RT planning and auto segmentation, advancing these algorithms for broad, clinically meaningful use requires addressing limitations in this workflow. One of the main limitations is due to the diffuse infiltration of glioblastoma, which leads to ambiguous tumor margins that make it difficult to distinguish between diseased and healthy tissue [11]. Standard RT planning relies heavily on expert manual or semi-automated segmentation of tumor and edema margins using MRI, a process that is subjective due to the heterogeneity of glioblastoma [12]. This subjectivity is worsened by inconsistent imaging techniques between institutions and interpretational differences among clinicians, leading to significant variability in tumor delineation and RT field definition. This complicates accurate tumor segmentation and classification, hampering both clinical decision-making and the development of reliable AI models. Therefore, the quality and consistency of ground truth used for training DL models are limited, impacting model generalizability and performance. 

In an effort to create consensus contouring guidelines for glioblastoma, a panel of 10 academic radiation oncologists specializing in brain tumor treatment contoured CTVs on four glioblastoma cases independently before convening to review their contours [13]. Variations across these experts spanned from the definition of T1C and T2-FLAIR signals (with kappa statistics of just 0.69 and 0.74, respectively) to expansions based on delineation of barriers to spread and preferred anatomic pathways of spread. Similarly, in re-irradiation settings, it is extremely challenging to define the extent of disease when tumor recurrences arise in a milieu of radiation necrosis (or treatment effect). Despite careful clinical annotation, training of models can easily be distorted by outliers in datasets [14], imposing costs on either target coverage and/or normal tissue sparing. Closely mimicking the challenges with standardization of RT treatment volumes is that of variability in imaging techniques. Not only do hardware (vendor, magnetic field strength and gradients, receiver coil geometry, etc.) and software (sequence acquisition and reconstruction algorithms) matter, but also deformation correction, intensity normalization, and validation of robustness, saliency, and sensitivity of models generated from such imaging datasets require close attention and careful benchmarking.

Furthermore, conventional RT approaches are largely static, utilizing only pre-treatment imaging as the basis for planning and failing to account for the dynamic changes that can occur during the course of and in response to therapy. Tumor volumes, peritumoral edema, and treatment-induced effects such as necrosis or pseudoprogression can alter the landscape of the tumor significantly, yet standard RT protocols are not designed to adapt to these temporal variations [15]. This can result in either under-treatment, where areas of true tumor progression fall outside of the planned target volume, or over-treatment, where unnecessary radiation is delivered to normal tissue. This can ultimately lead to post-treatment cognitive dysfunction in 30–50% of patients 6 months after RT [16]. Lastly, the growing availability of molecular data, such as transcriptomics and epigenomics, has not yet been fully incorporated into clinical workflows for RT planning. This data would provide valuable insights into tumor heterogeneity and treatment response, but the practical constraints of integrating large quantities of genomics data into RT planning still represent a critical gap in the field.

## 4. Tumor Delineation and Auto Segmentation

CNNs represent the most popular methods for auto segmentation; simplified inputs and outputs for such models are illustrated in Figure 1. This architecture and its variants have largely been considered state-of-the-art in computer vision and medical image processing for the last decade [17]. A variety of optimized CNN models (i.e., DeepMedic, ResNet, Seg-Net, etc.) are publicly available and serve as the initial building blocks in many of the studies outlined in this paper [18,19]. The most popular CNN architecture for auto segmentation is U-Net, a model which leverages an encoder–decoder framework for both down sampling and up sampling of data to preserve local and global image characteristics, thereby reducing noise while enhancing pertinent structural features [17]. Several commercial solutions for organs at risk segmentation also rely on similar technologies [20].

Performances of such models are standardized and compared primarily using the Dice similarity coefficient (DSC), which effectively calculates the spatial overlap between two sets of data, such as segmentations, normalized by sum of elements comprising both sets, such as total pixels occupied by the segmentations. A score of 1 designates perfect overlap between the predicted and ground truth volumes, which are typically manual segmentations or clinically approved auto segmentations, and 0 indicates no overlap [21].

The most common challenges for training and deploying CNNs include lack of large, annotated datasets of high quality and reproducibility. This has been addressed in recent years with large consortia of imaging data, such as the annual Medical Image Computing and Computer-Assisted Intervention Society Brain Tumor Segmentation (BRATS) challenge. This international dataset consists of shared multimodal MRIs, annotations, clinical outcomes, and expert-generated segmentations for three subregions: complete tumor, core tumor, and enhancing tumor. The BRATS challenge has played a critical role in advancing glioblastoma auto segmentation models over the past decade [22]. Early attempts of Medical Image Computing and Computer Assisted Intervention Society 2012 and 2013 BRATS challenge involved 20 algorithms trained on 65 multi-contrast MRI scans from both low- and high-grade glioma patients, with DSC performance ranging from 0.74 to 0.85 [22,23]. At that time, deep learning methods had not yet been re-established as state-of-the-art technology for segmentation and were not in use, as generative probabilistic methods were heavily favored [22]. As of 2018, clinicians still outperformed auto segmentation models, largely due to the limited size of annotated datasets available for training [24]. Despite this, CNNs soon became the leading architecture for segmentation and treatment planning by BRATS organizers in both 2017 and 2018 [25,26]. Since then, BRATS has continued to advance state-of-the-art computer vision for auto segmentation of gliomas. The 2021 benchmark pooled preoperative MRI data stacks from 2040 patients across multiple institutions. They also introduced the methylated-DNA-protein-cysteine methyltransferase methylation status challenge, inviting participants to train and validate radiogenomic predictions across a diverse clinical dataset [27]. In 2022, the winning ensemble primarily leveraged existing frameworks such as DeepSeg, DeepSCAN, nnU-Net, a novel self-configuring method that can reportedly be trained and deployed on a variety of different auto segmentations [28]. For whole tumor, enhancing tumor, and tumor core, the DSCs were 0.93, 0.88, and 0.88, respectively, on BRATS testing dataset [29]. A similar ensemble was developed in 2023, again using the popular nnU-Net framework; notably, the model also implemented data augmentation by generating synthetic MRI training data using generative adversarial networks. This approach was found to mitigate class imbalances with the addition of numerous unique tumor locations and compositions, allowing for high generalizability and DSCs of 0.90, 0.85, and 0.87 for whole tumor, enhancing tumor, and tumor core [30]. Most recently, the BRATS 2024 challenge placed increasing emphasis on post-treatment, including annotated resection cavity, non-enhancing tumor core, and non-enhancing T2/FLAIR hyperintensity. For this task, Ferreira, Moradi, and colleagues developed the top-performing model, once again using their nnU-Net synthetic data ensemble [31]. 

In recent years, DL models have continued to advance, offering improved precision, speed, and reproducibility in tumor-delineating auto segmentation. Numerous studies have since evaluated various CNN-based architectures (Table 1), including cascaded 3D Fully CNNs [32], hybrid ensemble models such as Incremental XCNet [33], and artificial neural networks validated on large institutional and public datasets [17]. Tools like AutoRANO demonstrated near-perfect intraclass correlation for volumetric tumor metrics using U-Net-based architectures [34]. One retrospective study trained a CNN model using diffusion tensor imaging to predict microscopic tumor infiltration margins, aligning with standardized guidelines for CTV delineation [7]. A recent multi-reader study found that deep neural networks reduced inter-reader variability and segmentation time in stereotactic radiosurgery planning compared to manual expert contours [21]. Architectural innovations, including attention-enhanced CNNs [35], densely connected micro-block Fully CNNs [36], holistically nested neural networks [37], and multiple U-Net variants [38], have achieved high DSCs while reducing processing time to mere seconds in some cases. Earlier studies using BRATS datasets also explored strategies like kernel optimization [39], cascaded inputs [40], multimodal MRI integration [41], and dual-patch batch normalization techniques [42], all contributing to improvements in segmentation accuracy and model efficiency. A recent top performer called PKMI-Net was developed to automate segmentation of GTV, high-dose CTV, and low-dose CTV using non-contrast CT, multisequence MRI, and medical record inputs. The model was trained on 148 patients across four institutions and tested on 11 cases with histologically suspected glioblastomas. PKMI-Net achieved DSCs of 0.94, 0.95, and 0.92 for GTV, high-dose CTV, and low-dose CTV, respectively, resulting in an overall DSC of 0.95. All outputs were deemed clinically acceptable without requiring revision. The architecture used a two-stage U-Net framework where the initial GTV segmentation informed subsequent high-dose CTV and low-dose CTV predictions, improving contextual accuracy across planning volumes [43].

In conclusion, DL continues to show strong potential for automating segmentation, margin detection, and RT planning. Advances in autoencoders and multi-layer CNNs have largely supplanted fully connected architectures. Many recent models are capable of automatically segmenting tumors with processing times as short as 20 s per patient [9]. Further, novel architectures, particularly diffusion models, are being investigated and show promise by coupling segmentation maps with uncertainty quantification. Nonetheless, high-quality labeling and collaborative data sharing remain essential to advance these technologies toward clinical implementation [9].

## 5. Personalized and Biologically Informed Tumor-Progression Radiotherapy

In conventional glioblastoma RT, the PTV is generally defined by applying an isotropic expansion to the CTV. This expansion is intended to account for potential errors in target delineation, setup uncertainties, and patient motion, thereby ensuring adequate dose coverage of the tumor. Such CTV approximation can result in centimeter-level errors in PTV definition, limiting treatment accuracy and increasing radiation exposure to healthy tissues [44]. Because tumor boundaries are difficult to define, clinicians often apply binary dose escalation protocols, maximizing dose to the core while minimizing dose to surrounding areas, even though microscopic infiltration frequently extends beyond visible margins [45].

A large-scale modeling study used data from 124 glioblastoma patients in The Cancer Genome Archive alongside 397 from the UCSF Glioma Dataset to identify relations between tumor proliferation, infiltration, and molecular pathway activation. A patient-specific growth model was created using contrast-enhanced T1 and T2/FLAIR MRI inputs, outputting tumor growth predictions within 4–7 min. The model was validated on 30 patients by comparing predicted recurrence volumes with those defined by standard radiation oncology practices. Findings reinforced that many recurrences occur beyond standard CTV margins, emphasizing the need for biologically grounded treatment planning. Deployable models must be time-efficient, highly validated, and compatible with clinical computational infrastructure to support practical integration [45]. As an extension of this concept, imaging-derived tumor sub-regions or spatial habitats can be computationally extracted from the relative intensities of pixels within multi-parametric datasets (say, T1, T1C, T2, and FLAIR) and correlated with genomic and molecular features [46]. In principle, these imaging correlates derived from larger MR sequence datasets could populate an assortment of signatures that map to each of the hallmarks of cancer [47], such that personalized interventions can be tailored to specific pathways and/cellular processes. From an RT standpoint, however, the identification of tumor sub-regions harboring inherently aggressive phenotypes (biological target volumes) may enable a degree of rational personalization of RT treatment volumes, expansions, and doses that improves upon what is currently available.

Tumor infiltration beyond the visible imaging margins could be more effectively accounted for through the integration of tumor growth models, which not only capture the spatial-temporal dynamics of glioblastoma progression but also enhance the biological interpretability of treatment planning. The development of such informed and personalized RT frameworks has the potential to derive these insights by integrating personalized tumor dynamics and multimodal imaging. A recent study outlined the use of a Bayesian ML model to infer tumor cell density using a reaction-diffusion model based on the Fisher-Kolmogorov equation [48]. This model incorporated preoperative MRI and FET-PET imaging to estimate microscopic infiltration beyond MRI-visible regions. In a clinical population study, the personalized RT plans derived from these inferred tumor densities showed comparable tumor coverage to standard RT while sparing more healthy tissue. Furthermore, the regions of high tumor cell density aligned with known radioresistant areas, suggesting that such biologically guided maps could inform dose escalation strategies. This approach demonstrates the feasibility of individualized treatment design with clinically available imaging modalities; however, external validity and generalizability are questionable given a small testing cohort of just 8 patients [48]. Although such a model is favorable to DL approaches in terms of explainability and biological interpretability, there are often tradeoffs to performance and interpretability that limit the utility compared to winning models in the annual BRATS challenge [24].

PET imaging is less emphasized in our review, primarily given the fact that most of the cited models were trained and validated using CT- and MRI-based modalities. This likely directly reflects the data included within the annual BRATS challenge, which has never included PET imaging data. However, the relative potential impact for such spatially localizing, metabolically informative imaging should not be understated, as it is vitally important for management of glioblastoma [49]. Thus, if PET is to be effectively integrated into existing DL frameworks, it is crucial to create large, shared consortia in addition to corresponding patient CT and MRI.

## 6. Modification of Treatment, Patient Response Prediction, and Triage During Therapy

Despite advances in RT techniques, clinical outcomes for many patients with glioblastoma remain poor, underscoring the limitations of current treatment strategies. Timely identification of patients at heightened risk for unfavorable outcomes during the course of RT is essential for improving therapeutic efficacy, reducing the likelihood of treatment interruptions, and mitigating the associated burden on healthcare systems. ML offers a transformative opportunity in this regard, as it enables the systematic integration and analysis of large-scale, multimodal clinical datasets. By uncovering complex patterns that are often indiscernible to conventional statistical approaches, ML-based models have the potential to support early risk stratification, guide adaptive treatment strategies, and ultimately contribute to more personalized and effective patient care [50]. Efforts to characterize local tumor infiltration, predict dose distributions based on individualized anatomy and prescription, and ultimately forecast overall clinical outcomes have gained increasing attention in recent years [8].

A recent multi-institutional study utilized leave-one-out cross validation to train and evaluate a patch-based CNN using multi-parameter MRI data stacks from 229 glioblastoma patients to predict regions of interest as either high- or low-infiltration. According to their findings, patients were found to be 8.13 to 19.48 times more likely to experience tumor recurrence when having high-infiltration regions compared to low-infiltration regions [51]. This highlights the requirement for datasets to be annotated by specific regions. DL is only as good as the data it is trained on, and its continued improvement and novel insights could potentially be catalyzed by further stratification of tumor-infiltrating regions. DL has also been used in research to rapidly segment patient images. DeepMedic was applied retrospectively to assess high-grade glioma recurrence, offering segmentation-derived insights [18]. The study suggested that reirradiation is safe and effective in glioblastoma treatment, showing the utility of auto segmentation models in treatment planning and optimization.

A large clinical study evaluated the effectiveness of an ML-based triage system utilizing electronic medical record data for predicting acute care needs during RT and chemoradiation. The algorithm assessed 963 outpatient adult RT or chemoradiation treatment courses to identify patients with a ≥10% risk of requiring acute care, defined as emergency department visits or hospital admissions during treatment. Of these, 311 courses were randomized to either standardized weekly or required biweekly clinical evaluations. Patients identified as high-risk by the ML model and assigned to the intensified clinical follow-up experienced a significant reduction in acute care visits, dropping from 22.3% to 12.3% compared to those receiving standard care (*p* = 0.02). The model demonstrated strong predictive value, with a receiver operating characteristic area under the curve (AUC) of 0.851, supporting its potential as a tool for real-time patient management during therapy [52].

In a separate study focused on treatment response prediction, ML was used to stratify patients undergoing RT as likely responders or non-responders from radiomic features. Response probability was output by the model and compared to clinician assessments. A decision threshold of 67% was set by the model to classify patients as responders versus non-responders. The model achieved an accuracy of 75% with an AUC of 0.74, outperforming clinician assessments, which achieved an accuracy of 54% and an AUC of 0.56. These findings underscore the ability of ML frameworks to integrate complex imaging and clinical data to outperform physician-predicted therapeutic response [53]. Integrated end-to-end workflows have also been developed. One example combined automated glioblastoma segmentation and ensemble-based survival prediction into a single pipeline trained on BRATS-2020 data. The model classified patients as long (>12 months) or short (<12 months) term survivors with AUCs of 0.86 and 0.72 on BRATS-2020 and institutional datasets, respectively. The auto segmentation component achieved a DSC of 0.91, supporting the model’s utility in streamlining the entire planning process from segmentation through prognosis [50]. One study further demonstrated the potential for mapping dose escalation and demonstrated PTV coverage comparable to manual segmentation, though training and testing were greatly limited due to small sample sizes of 95 and 15 patients, respectively [54].

Together, these studies highlight how AI-guided tools can assist not only in pre-treatment planning but also in monitoring and adjusting care during the treatment course [55]. By identifying patients who may benefit from closer follow-up or alternative treatment strategies, these models offer the potential to improve clinical outcomes, reduce treatment-related complications, and optimize healthcare resource utilization.

## 7. Radiogenomics and Non-Invasive Biomarker Integration

Radiogenomics represents a growing intersection between imaging, ML, and genomic data, offering the potential to guide personalized treatment strategies in glioblastoma. This approach is particularly promising given the heterogeneity of glioblastoma, which limits the predictive utility of traditional histopathology and challenges the generalizability of fixed RT protocols.

Multiple ML frameworks have been employed to predict genetic mutations and classify glioma subtypes based on imaging features. One study trained a residual CNN model using 406 preoperative brain MRIs to predict isocitrate dehydrogenase mutation status, achieving a testing accuracy of 85.7% [56]. The ability to infer genotype from imaging suggests a reciprocal potential, where known mutation status could be integrated into AI models to inform RT planning [56]. This is especially relevant given that patients with isocitrate dehydrogenase-mutated glioblastoma have demonstrated significantly longer overall survival and progression-free survival compared to isocitrate dehydrogenase-wildtype cases (overall survival of 39 months vs. 14 months), independent of treatment status [57]. Similarly, methylated-DNA-protein-cysteine methyltransferase promoter methylation has been associated with increased responsiveness to alkylating agents, including temozolomide, highlighting the importance of genomic markers in therapeutic decision-making [19].

A random forest-based radiomics model was developed for glioma grading using contrast-enhanced T1-weighted MRI from a training cohort of 101 patients. Testing on an independent cohort of 50 patients from two external institutions yielded an AUC of 0.898, with 84% sensitivity, 76% specificity, and 80% accuracy. The highest-performing model combined DL features from a simple architecture CNN-based model, VGG16, with traditional radiomic features, outperforming either input modality alone [58]. In another study, support vector machine classification combined with synthetic minority over-sampling was able to differentiate high (grades 3 and 4) and low (grades 1 and 2) grade gliomas with 94–96% accuracy, further supporting the utility of hybrid AI approaches [59]. Recently, a DL model was cross validated on 357 patients with isocitrate dehydrogenase-wildtype glioblastoma using pre-operative multiparametric MRI. Notably, the model also incorporated radiogenomic features using genetic sequencing data, enabling spatial mapping of critical gene mutations, including NF1, TP53, PTEN, and EGFR. The multimodal framework was compared with an MRI-only CNN model as well as a radiogenomic-only support vector machine, outperforming both with an AUC of 0.70–0.92 across 13 different biomarkers [60]. This, along with the aforementioned studies, further bolsters the case for incorporating combined models that can leverage both imaging and heterogeneous, molecular-level precision. 

**Figure 2:**
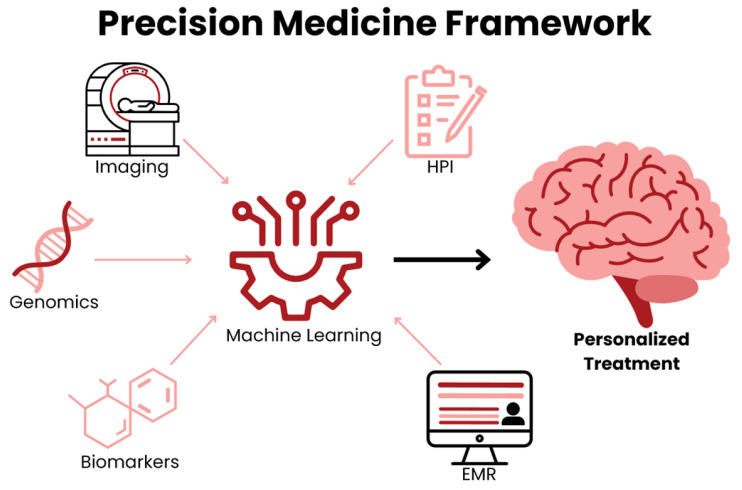
Personalized precision medicine for patient-specific radiotherapy treatment.

Beyond imaging, non-invasive biomarker techniques such as liquid biopsy are being explored for diagnostic and therapeutic monitoring purposes. Circulating tumor DNA and microRNA can provide insights into tumor status, although their reliability remains limited. Transport is impeded by the blood–brain barrier, and intratumoral phenotypic heterogeneity is masked by assessment of a cumulative metric diluted in the systemic circulation. Brain biopsy remains the gold standard but is invasive and carries sampling-related risks. Novel systemic immune-inflammation indices offer non-invasive alternative biomarkers to benchmark clinical grade, glioma subtype, and patient prognosis [61]. Additionally, serum levels of exosome microRNA, along with specific microRNA expression profiles (e.g., miR-21, miR-181c, miR-195, miR-196b), may serve as prognostic biomarkers to accurately predict glioma status and treatment outcomes [10,62,63,64,65]. Furthermore, the integration of radiogenomics with precision population cancer medicine offers a novel approach for comprehensive and longitudinal monitoring of patients, enhancing individualized care and treatment stratification (Figure 2). Initiatives like the Children’s Brain Tumor Tissue Consortium are advancing this goal by curating large-scale shared data repositories. Complementary bioinformatics tools, such as NetworkAnalyst, OmicsNet, Cytoscape, and Alphafold, facilitate exploration of protein–protein interactions, while multimodal approaches combining MRI, genomics, metabolomics, and AI imaging offer a multidimensional view of tumor biology [66].

Radiogenomics and non-invasive biomarker integration provide a promising framework for future glioblastoma treatment personalization. Continued expansion of multi-institutional datasets, model validation across diverse patient populations, and incorporation of comprehensive molecular profiles into imaging-based models will be essential for translating these technologies into clinical care. Modernized data stewardship practices and incentives for pooling population-scale data sets are a crucial step toward providing balance to data and a data ecosystem and models that are representative of populations [67,68]. Validated outcomes data and high-quality ground truth are also a significant hurdle to model validation when considering more nuanced outcomes beyond progression or disease survival [69].

A systematic review of 14 radiogenomics studies reported AUC values ranging from 0.74 to 0.91 but found no consistent patterns based on imaging modality. Modalities used across the studies included T1, T1C, T2, FLAIR, DTI, DWI, spectroscopy, Dynamic Susceptibility Contrast, and Dynamic Contrast Enhanced-MRI. AI techniques included support vector machine, diagonal linear and quadratic discriminant analysis, semi-supervised learning, and CNNs. All studies included MRI as a required input, and all models were trained on single-institution datasets with limited sample sizes (8–37 patients), increasing the risk for protocol-specific biases and overfitting [70]. It is worth noting that the real-world impact of these models on patient survival is unknown, as they have yet to be implemented into clinical practice.

## 8. Interpretability and Explainability in Deep Learning Models

DL models, often referred to as “black-box” systems, have demonstrated impressive performance in RT planning for glioblastoma; however, a key barrier to clinical adoption remains the lack of consistent performance and generalization. This is further complicated by the absence of interpretability and explainability of these models, in particular when determining treatment plans and responses [23,71]. For example, the challenge of inter-institutional variability was demonstrated in a CNN model trained on 44 glioblastoma patients across two institutions. When tested within the same institution, DSCs reached 0.72 and 0.76. However, when validated across institutions, performance dropped to 0.68 and 0.59, highlighting the need for large, diverse datasets to develop models capable of generalizing across clinical settings [72]. Beyond dataset size, sources of bias such as heterogeneity in imaging protocols, scanner physics, operator technique, patient demographics, and post-processing software must also be considered. Incorporating harmonization strategies and bias-aware model training will therefore be as critical as expanding dataset diversity in addressing systematic variability.

Normally, the most accurate methods, such as DL, are the least transparent, while methods encouraging transparency, such as decision trees, are less successful in their performance [73]. The explainability of AI systems is essential for fostering trust among medical professionals and could play a vital role in facilitating AI integration into clinical practice. Clinicians need a clear understanding of how automated algorithms arrive at specific segmentation, dose planning, or treatment response predictions in order to trust and effectively utilize these outputs in patient care [74]. Fortunately, the risks of unsupervised, black box predictions are inherently mitigated by rigorous assessment of dose maps by the RT team with adherence to strict Radiation Therapy Oncology Group guidelines for glioblastoma, given that physicians can simply adjust the auto-generated plans as necessary. However, lack of transparency poses a significant challenge for incorporation of predictions, such as treatment response, which cannot be readily verified by physicians. While interpretability is important, precedent from genomic clinical decision support shows that black-box algorithms can still clear regulatory hurdles and achieve widespread use when backed by strong evidence that they accomplish the intended purpose. Thus, emphasis should be placed on rigorous prospective validation, unbiased benchmarking, and tools such as Shapley value plots providing insight into model reasoning in the absence of full explainability.

Saliency mapping approaches, including class activation mapping, gradient-weighted class activation mapping, and integrated gradients, have been introduced to generate post hoc explanations of model predictions [65,75,76]. For example, gradient-weighted class activation mapping overlays gradients or heatmaps of any target concept, such as tumor segmentation, on MRI or CT images, transitioning into the final convolutional layer to output a spatial map that visually indicates which localized regions most influence the target concept. These visualizations not only enable clinicians to verify that AI is focusing on clinically plausible anatomic patterns but also flag instances where the model may be inappropriately influenced by artifacts or non-tumor structures. Furthermore, these visualizations lend insights when models fail and help achieve model generalization by identifying dataset bias.

Other methods, namely Shapley additive explanations and Local Interpretable Model-Agnostic Explanations, are perturbation-based and model-agnostic, requiring only model input and output [77]. For example, if a model predicts that a patient has a high risk of recurrence, Shapley additive explanations assign each feature of a dataset (tumor size, patient age, treatment history, etc.) an importance value for a particular prediction to show how much each factor contributed to that prediction. The model works by analyzing many combinations of these features and distributing the influence each one has on the final output. While these methods have been widely used for tabular or radiomics data, emerging studies are adapting Shapley additive explanations values to highlight influential image features or radiomic descriptors relevant to segmentation boundaries or radiogenomic predictions.

Despite significant promise, several risks are still associated with some of the proposed AI-assisted RT workflows. Namely, overfitting persists as a pertinent issue, especially when models are trained on limited or single-institution datasets. Models that appear to be top performers during validation may hold poor external generalizability when applied to unseen data, potentially leading to inaccurate target volumes. Such errors might lead to underdosing infiltrative tumor margins. Perhaps the most pressing challenge lies in automation bias: the inherent tendency to gravitate towards the AI-generated dose map, specifically when physicians are faced with time constraints or when the model has exceedingly high historical performance. Thus, it is imperative to maintain rigorous verification based on standardized RT guidelines.

Recent research also explores uncertainty quantification in model outputs, either through Bayesian DL or using Monte Carlo dropout during inference. This produces probabilistic segmentation maps or confidence intervals for dose predictions, supporting clinicians in assessing which automated outputs warrant further scrutiny or consensus review. In a recent study, two Bayesian DL models were assessed alongside eight uncertainty measures, utilizing 292 PET/CT scans comprising a sizeable cross-institutional dataset to investigate their RT approach for oropharyngeal cancer treatment. The study accurately estimated the quality of their novel DL segmentation in 86.6% of cases; more importantly, however, it successfully recognized areas of interest and cases where the DL framework generated low certainty and therefore increased probability of poor performance [78]. 

Overall, there is a push to integrate these explainability tools into AI-based RT planning software as standard features. Transparent outputs allow for cross-checking, identify hidden model biases, and reveal avenues for model optimization and re-training, thereby increasing the likelihood of clinician uptake.

Although interpretability and model performance are crucial for clinical deployment, the importance of ethical and regulatory considerations cannot be understated. Data privacy remains a significant challenge given the utility of multimodal data, including imaging, patient history, genomic profiles, etc. Further, multi-institutional data sharing adds another layer of complexity in transmitting anonymized patient data while maintaining high-quality annotated labels. Clear data sharing guidelines must be established and routinely audited to ensure strict HIPAA compliance. Liability and clinical responsibility also become a concern when integrating AI within any clinical decision-making workflow. Physician documentation is crucial in establishing transparency, as responsibility for the output segmentation ultimately lies in the hands of the care team.

## 9. AI-Driven Solutions and Current Trends in Technology

While CNNs remain the standard for RT planning in glioblastoma, alternative AI-driven solutions are also being developed to address the challenges in the current workflow. For example, intraoperative imaging combined with AI-driven segmentation could enhance tumor boundary detection in real time. In RT planning, DL models trained on consensus-derived contours could standardize target definition and reduce inter-observer variability. Real-time multimodal imaging can be combined with DL models to predict tumor trajectory and adjust treatment dynamically. ML applied to multi-omics data could help further characterize tumors, helping guide RT planning and treatment personalization. Such integrative approaches could pave the way for AI-driven, personalized RT planning that addresses the current challenges that physicians face in glioblastoma management and treatment.

CNNs are limited by their reliance on local receptive fields to segment tumors with poorly defined or infiltrative margins, but recent advances have incorporated self-attention mechanisms and transformer-based architectures to capture long-range dependencies and contextual information, improving boundary delineation [79]. Additionally, consensus learning frameworks can integrate outputs from multiple models or annotators to reduce interobserver variability and enhance segmentation reliability [80]. DL-based harmonization and normalization techniques can also minimize the impact of scanner and protocol-related variability, enhancing reproducibility across institutions [81]. Collectively, these approaches address one of the key limitations of traditional CNNs and represent an important step toward AI-driven tumor segmentation.

Another promising solution involves integrating multi-omics data into ML frameworks to guide RT planning and improve tumor classification. For example, the integrative glioblastoma subtype classifier leveraged both gene expression (transcriptomic) and DNA methylation (epigenomic) data in a multi-omics model [82]. Using Random Forest for feature selection and Nearest Shrunken Centroid for classification, integrative glioblastoma subtype classifier achieved a high mean AUC of 0.96, outperforming classifiers built on either data modality alone. The authors utilized only five features per subtype and were able to produce a highly accurate, cost-effective model from large-scale genomics data. This approach provides a template for merging multi-omics data with imaging-driven predictions to enhance tumor classification and segmentation accuracy. This can allow for patient-specific risk stratification and dose personalization, especially as the accessibility of high-throughput molecular testing improves.

Next, adaptive radiotherapy accounts for temporal changes in tumor position, volume, and response over the course of treatment. Through multimodal imaging with PET or MRI, adaptive radiotherapy captures high-resolution datasets to precisely evaluate anatomical changes in tumor shapes, borders, and locations throughout treatment [83,84]. State-of-the-art systems can quickly process real-time imaging data and even optimize radiation beam placement and intensity, making in-session adjustments to the treatment plan and accommodating daily variations in the patient’s anatomy [85]. Continuous tracking offers real-time feedback, enabling rapid correction if there are any unexpected factors or significant deviations from the original treatment plan. A study by Guevara et al. studied whether adaptive radiotherapy could reduce the RT dose with the aim of improving post-RT cognitive function [86]. They evaluated 10 glioblastoma patients who previously received RT treatment over six weeks without adaptation and simulated weekly plans that adjusted the dosage according to the shrinking tumor. While still targeting the cancerous tissue, the mean and maximum doses administered to the hippocampus and brain were significantly reduced for the adjusted plan. Therefore, incorporating adaptive radiotherapy into pre-existing CNN architectures can address the limitations of static treatment, possibly mitigating the neurocognitive side effects of RT for patients.

Lastly, the emergence of foundation models and latent diffusion architecture represents the latest trends in AI for glioblastoma treatment. Foundation models are large-scale models pre-trained on millions of images and require fewer labeled examples, and demonstrate improved standardization and data-efficiency [87]. For example, the Segment Anything foundation model, trained only on object segmentation in 2D photographs, was given the BRATS challenge and achieved high accuracy for interactive glioma MRI segmentation [88]. In parallel, latent diffusion models generate 3D multi-modal images of brain MRIs and their corresponding masks to augment scarce datasets. Diffusion models are trained by adding noise to an image in a series of iterative steps, gradually denoising, and transforming a noise vector into an image. This methodology allows them to capture complex, high-dimensional structures and generate synthetic images from the underlying data, boosting both the quantity and quality of training data for complex tasks like tumor segmentation [89]. These generative models underpin newer pipelines for auto segmentation and synthetic-CT/field optimization, complementing foundation models and feeding downstream dose-prediction networks. Together, these solutions highlight the growing role of DL in advancing and redefining the glioblastoma treatment workflow.

## 10. Conclusions

Deep learning models have the power to dramatically streamline clinical workflow and are already being deployed to do so. Manual segmentation is redundant, tedious, and time-consuming for physicians, and convolutional neural networks offer the perfect framework to rapidly automate this task with minimal risk due to standardized treatment guidelines and strict interprofessional review boards. In contrast, the outlook for treatment response prediction and personalized artificial intelligence-generated therapy regimens remains uncertain due to the necessity for substantial clinical data, likely requiring randomized clinical trials to ensure patient safety and efficacy if any changes are to be made to treatment guidelines. Still, deep learning can dramatically optimize clinical workflow with auto segmentation, offering physicians the ability to spend more time with their patients where their time matters most.

Cross-institutional data annotation, sharing, and validation are crucial steps toward improving the performance and generalizability of deep learning models for glioblastoma radiotherapy planning. These collaborative efforts account for inter-institutional variability in imaging acquisition protocols, hardware, and preprocessing methods, which otherwise increase the risk of model overfitting. This point is made clear by comparing internal validation metrics presented in Table 1. For example, ref. [37] achieved similar performance to [32] despite training on data from 10 patients compared to 1652, respectively [32,37]. Obviously, the latter is more likely to perform favorably in cross-institutional validation due to greater data diversity and model generalizability. Thus, external validation should be thought of as mandatory for such models for benchmarking performance. By incorporating diverse datasets, models can be better adapted to highly variable clinical scenarios, including those in rural or resource-limited settings where imaging quality and technique may differ significantly. While larger academic hospitals have begun deploying deep learning models trained on proprietary datasets, broader collaboration is essential to ensure equitable access to high-quality, artificial intelligence-assisted care. Further, bifurcation within single institution practices and fragmentation between multiple institutions carry risk of lowering generalizability and external validity.

It is important to emphasize that, in the absence of prospective data validated by clinical trials, standard radiotherapy protocols established by the Radiation Therapy Oncology Group must remain the foundation of treatment planning. Such guidelines require 2 cm margins around visible tumor boundaries, and any artificially generated segmentation that reduces this margin would be subject to rigorous clinical review. As such, all segmentation outputs, regardless of whether they are clinician-derived or model-assisted, undergo final approval by the radiation oncology team, including the attending physician, dosimetrists, and medical physicists. Outputs must comply with established standards of care in the absence of compelling evidence substantiated by randomized controlled trials.

In addition to technical concerns, there are also critical workflow and human-factor limitations that arise once artificial intelligence systems are introduced into clinical practice. One key risk is automation bias, where clinicians may place excessive trust in artificially generated contours or treatment plans, potentially accepting results without sufficient review [90]. This becomes especially problematic when the system’s output appears precise but lacks contextual nuance or clinical appropriateness. Poorly designed user interfaces can further amplify this issue by making artificial intelligence tools difficult to navigate or inefficient to use, which can lead to passive acceptance rather than informed engagement. Even when models perform well in controlled environments, they may still fall short of being clinically acceptable if they are not designed to integrate smoothly into real-world workflows, support clinician autonomy, and promote active decision-making. A concise overview of the pros and cons of artificial intelligence-based methods vs. manual methods is provided in Table 2.

Deep learning methods have served as state-of-the-art architecture in medical computer vision for over a decade. Numerous studies have demonstrated their effectiveness in tumor segmentation, radiogenomic prediction, and dose planning. Vision transformers are now emerging as a disruptive alternative to auto segmentation models, with growing evidence suggesting their ability to outperform convolutional neural networks due to their improved contextual understanding across spatially distant regions of an image. A significant limitation of vision transformers is their high data demand, requiring expansive, high-quality annotated datasets. As larger annotated imaging datasets become available through shared institutional repositories and as data augmentation techniques continue to evolve, vision transformers are likely to emerge as a viable competitor for auto segmentation in the coming years. This transition will hinge on continued investment in data infrastructure and collaborative research networks. 

Ultimately, the integration of biologically informed mathematical modeling, multimodal imaging, and genomic data represents a significant evolution in glioblastoma radiotherapy. By capturing the heterogeneity of tumor infiltration and biological behavior, these frameworks can enhance tumor targeting, minimize toxicity, and advance traditional approaches that have failed to improve patient survival for decades [41]. In parallel, coordinating standards for data curation and artificial intelligence validation within radiation oncology and neuro-oncology communities will be essential for clinical translation. Organizations such as the American Society of Clinical Oncology’s information technology and artificial intelligence initiatives, the National Cancer Institute’s Imaging Data Commons, and the American Society for Radiation Oncology are well-positioned to serve as connective tissue between academic medical centers and large practice groups. Establishing such a formalized community of practice could accelerate adoption, minimize redundancy, and ensure that advances in artificial intelligence for glioblastoma radiotherapy are both reproducible and clinically actionable.

## Figures and Tables

**Table 1:** Summary of DL models for auto segmentation of brain tumors from 2016-2025.

Author	Year	Study/Model	Input Data	Patients/ Plans	Performance (DSC)	Notable Features
Peeken et al. [7]	2019	CNN	DTI; FLAIR	33	NA	Microscopic infiltration mapping aligned with clinical RT guidelines
Lu et al. [21]	2021	3D U-Net + DeepMedic Ensemble	CECT; T1C	1288	0.86–0.90	Real-time clinical SRS workflow integration
Xue et al. [32]	2020	Cascaded 3D FCN	3D-T1-MPRAGE	1652	0.85	High-volume validation
Naceur et al. [33]	2018	Incremental XCNet	T1C; T1; T2; FLAIR	210	0.88	Novel parallel CNNs + ELOBA_λ training; 20.87 s/segmentation
Kickingereder et al. [17]	2019	ANN	T1C; T2	455	0.89–0.93	Segmentation output < 1 min; longitudinal validation
Chang et al. [34]	2019	AutoRANO (U-Net)	T1C; FLAIR	843	0.94	Outputs RANO metrics for volumetric tracking
Ranjbarzadeh et al. [35]	2021	Cascaded CNN w/Attention	T1; T1C; T2; FLAIR	285	0.92, 0.87, 0.91 *	Reduced training time by 80%
Deng et al. [36]	2019	FCNN + DMDF	T1; T1C; T2; FLAIR	100	0.91	Segmentation output < 1 s
Zhuge et al. [37]	2017	HNN	T1; T1C; T2; FLAIR	10	0.83	Weighted-fusion; 10 h training
Isensee et al. [38]	2018	Modified U-Net	T1C; T1; T2; FLAIR	220	0.90, 0.80, 0.73 *	Top performer that year
Pereira et al. [39]	2016	CNN (3 × 3 kernel)	T1C; T1; T2; FLAIR	65	0.88, 0.83, 0.77 *	BRATS-2013 top performer & BRATS-2015 2nd overall
Havaei et al. [40]	2017	Cascaded CNN (2nd DNN input)	T1C; T1; T2; FLAIR	65	NA	Reduced segmentation time by 30-fold
Soltaninejad et al. [41]	2017	RF + FCN	T1C; T1; T2; FLAIR	65	0.88, 0.80, 0.73 *	RF & FCN ensemble
Hussain et al. [42]	2018	Deep CNN w/dual-patch input	T1C; T1; T2; FLAIR	274	0.87, 0.89, 0.92 *	Novel batch normalization
Tian et al. [43]	2024	3D U-Net	NCCT; T1C; FLAIR	148	0.92, 0.87, 0.91 *	Novel two-stage 3D U-Net
Moradi et al. [31]	2025	nnU-Net ensemble	T2; FLAIR	150	0.83	Synthetic training data; BRATS-2023 and 2024 top performer

* Whole tumor, tumor core, enhancing tumor, respectively; T1: T1-weighted MRI; T2: T2-weighted MRI; T1C: T1-weighted MRI with contrast; FLAIR: T2 fluid-attenuated inversion recovery MRI; CECT: Contrast enhanced computed tomography; NCCT: Non-contrast computed tomography; RANO: Response assessment in neuro-oncology; SRS: Stereotactic radiosurgery; 3D-T1-MPRAGE: Three-dimensional T1-weighted magnetization-prepared rapid gradient echo; DWI: Diffusion-weighted MRI; NA: results and full text not publicly available

**Table 2:** Summary of pros and cons of manual vs. AI-based radiation treatment planning.

	Manual		Artificial Intelligence	
**Auto-segmentation**	**PROS**	- Physician-driven	**PROS**	- Faster segmentation
		- Context-aware decisions		- Improved consistency
		- Customizable per patient		- Scalable across cases
	**CONS**	- Time-consuming	**CONS**	- May miss subtle anatomical nuances
		- Intra-/inter-observer variability		- Limited generalizability
		- Inconsistent delineation standards		- Requires clinician verification
**Dose planning**	**PROS**	- Greater human oversight	**PROS**	- High-speed prediction
		- Flexible for complex cases		- More standardized plans
				- Learns from large datasets
	**CONS**	- Labor-intensive	**CONS**	- May not account for patient-specific anatomy
		- Less reproducible		- Potential overfitting to training data
		- Susceptible to planning variability		
**Biologically informed RT**	**PROS**	- Direct clinical judgment in biomarker relevance	**PROS**	- Can integrate complex genomic/radiomic data
		- Custom-tailored escalation decisions		- Identifies non-obvious dose–response patterns
	**CONS**	- Limited by available validated biomarkers	**CONS**	- Models may lack transparency
		- Not easily reproducible across institutions		- Biological relevance not always clinically validated
**Treatment response prediction**	**PROS**	- Based on clinician experience and medical history	**PROS**	- Detects hidden correlations
				- Potential for early outcome forecasting
	**CONS**	- Subjective and inconsistent	**CONS**	- Risk of bias
		- Cannot scale or track subtle data patterns		- Generalizability across cohorts is limited
**Radiogenomics integration**	**PROS**	- Precision when available	**PROS**	- Merges imaging and genetic data
		- Personalized to patient		- Hypothesis-generating at population level
	**CONS**	- Not feasible at large scale	**CONS**	- Often exploratory
		- Requires multidisciplinary interpretation		- Lacks consistent clinical validation
**Interpretability/Decision Support**	**PROS**	- Transparent reasoning	**PROS**	- Synthesizes multimodal data
		- Based on clinical logic		- Suggests patterns not obvious to clinicians
	**CONS**	- May overlook complex data relationships	**CONS**	- Often black-box models
		- Hard to scale to multi-omic inputs		- May reduce clinician trust if unexplained

## Abbreviations

AI	Artificial Intelligence
RT	Radiotherapy
CTV	Clinical Target Volume
GTV	Gross Target Volume
PTV	Planning Target Volume
DL	Deep Learning
CNN	Convolutional Neural Network
ML	Machine Learning
DSC	Dice Similarity Coefficient
BRATS	Brain Tumor Segmentation
AUC	Area Under the Curve
